# Noise Floor Reduction in Frequency Delta-Sigma Modulation Microphone Sensors

**DOI:** 10.3390/s21103470

**Published:** 2021-05-16

**Authors:** Koichi Maezawa, Masayuki Mori, Hiroya Andoh

**Affiliations:** 1 Faculty of Engineering, University of Toyama, 3190 Gofuku, Toyama 930-8555, Japan; morimasa@eng.u-toyama.ac.jp; 2 Department of Information and Computer Engineering, National Institute of Technology, Toyota College, 2-1 Eiseicho, Toyota 471-8525, Japan; andoh@toyota-ct.ac.jp

**Keywords:** delta-sigma modulation, frequency delta-sigma modulation, microphone, microwave oscillator, phase noise, cavity resonator, FPGA

## Abstract

Frequency delta-sigma modulator (FDSM) employing a variable frequency oscillator is a novel replacement of the classical delta-sigma modulators. This is advantageous for application to sensors, because an ADC can be intrinsically integrated with the sensors. We have already proposed to use this technique to various sensors. However, the signal-to-noise ratio was significantly degraded by noise floor, in the previous papers. In this paper, we have investigated the origin of the noise floor in the FDSM microphone sensors as a promising example. It was demonstrated that improving the phase noise of the oscillator can drastically reduce the noise floor. For this reduction we improved the Q-factor of the cavity resonator, and the design of the oscillator circuit. With these improvements, the phase noise, and, hence, the noise floor, were improved by approximately 40 dB. In addition, we obtained an SNR of 57 dB for 114 dBSPL sound input with 96 kHz bandwidth, which corresponds to the dynamic range of 87 dB for maximum 140 dBSPL. A much larger dynamic range of around 120 dB is expected by increasing the sampling rate and decreasing the Al diaphragm thickness. These results also indicate the promise of the FDSM to varieties of physical sensors.

## 1. Introduction

Recent progress in digital technology has increased the importance of an analog-digital converters (ADC). In particular, high performance sensors need high performance ADCs for sophisticated data analysis and data transfer. Among the various types of the ADCs, a delta-sigma modulation ADC is often used for high resolution applications. This is because the delta-sigma ADC has a significant advantage in that high resolution can be obtained without high-accuracy analog components [1,2,3]. The delta-sigma ADC consists of a delta-sigma modulator (DSM) and a digital filter. The DSM converts an input analog signal into one-bit pulse density modulated digital sequence (PDM) at a frequency that is much higher than the Nyquist rate. Subsequently, the filter converts the PDM into multi-bit digital signal at the Nyquist rate. The core component ruling the accuracy and resolution of the ADC is the DSM. The conventional DSM is composed of a one-bit sampler, an integrator, and a feedback digital-analog converter (DAC). Here, the integrator and feedback DAC restrict the operation speed of the DSM.

Recently, another type of the DSM, called Frequency Delta-Sigma Modulator (FDSM), has attracted considerable attention, which uses a voltage controlled oscillator (VCO) [4,5,6,7,8,9,10,11,12,13,14]. The FDSM uses an intermediate frequency modulation (FM) signal that is generated by the VCO, and it has no feedback DAC or integrator. Therefore, it is suitable for high frequency operation.

Moreover, the FDSM can also be used for digital output sensors when the VCO is replaced by the oscillator whose oscillation frequency depends on an external physical parameter. The FDSM sensors have significant advantages. First, a sensor can be integrated with an ADC, and it directly converts the physical parameter to digital signal. Therefore, it has fewer noise sources, and the performance is not restricted by the external ADC. Second, it is robust against the external noises. In addition, it has an inherent advantage that it can have a large dynamic range with a wide bandwidth, when using a high frequency oscillator with a high sampling ratio. Because the performance of FDSM sensors significantly depends on the oscillation frequency, using compound semiconductor high-frequency devices in the oscillator offers considerable advantages.

FDSM strain sensors and microphone sensors were recently proposed by employing a high electron mobility transistor or a resonant tunneling diode for a gain block of the variable frequency oscillators [15,16,17,18,19]. Basic operations have also been demonstrated using prototype devices. However, previous reports have often shown non-negligible noise floor restricting the dynamic range. The origin of the noise floor was discussed based on a simple model in the reference [20], and it was concluded that the phase noise of the oscillator causes the noise floor. Under these circumstances, the purpose of this paper is to experimentally demonstrate the reduction of noise floor in the FDSM sensors by improving the phase noise property of the oscillator. We will investigate the noise floor in the FDSM microphone sensors employing a cavity resonator for the oscillator [16] as a promising example. We will report on the demonstration of the noise floor reduction by reducing the phase noise of the oscillator with a sophisticated design.

This paper is organized, as follows. Section 2 explains the operating principle of FDSM. Section 3 introduces the FDSM microphone sensors using a variable frequency oscillator based on a cavity resonator. Section 4 describes the improved design of the oscillator for reducing the phase noise. The experimental results are shown in Section 5. Finally, Section 6 summarizes this paper.

## 2. Frequency Delata-Sigma Modulation

In this section, the structure and operating principle of the FDSM are discussed after a brief introduction of the delta-sigma ADC and the conventional DSMs.

### 2.1. Delta-Sigma ADCs and Delta-Sigma Modulators

**Figure 1:**
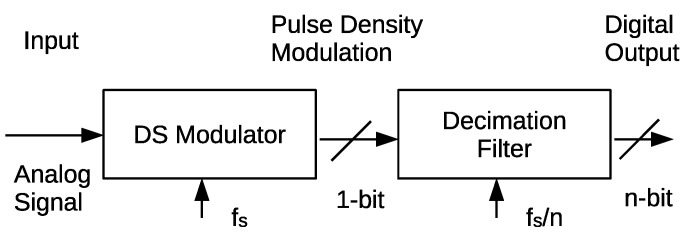
Block diagrma of the delta-sigma ADC.

Figure 1 shows a block diagram of the delta-sigma ADC. The delta-sigma ADC consists of a DSM and a decimation filter. The input analog signal is first converted to the PDM signal by the DSM at a sampling frequency, $f_{s}$, much higher than the Nyquist rate, $f_{N}=f_{s}/n$, here, *n* is an oversampling ratio. The quantization noise is reduced at low frequencies at the expense of its increase in high frequencies in this process. As a result, the quantization noise decreases with decreasing frequency by 20 dB/dec. This is called noise shaping. Finally, the decimation filter cuts the high-frequency noise component, and down-converts the 1-bit pulse density signal into the high-resolution digital output at the Nyquist rate. The in-band quantization noise is significantly reduced, owing to the noise shaping.

### 2.2. Frequency Delta-Sigma Modulators

**Figure 2:**
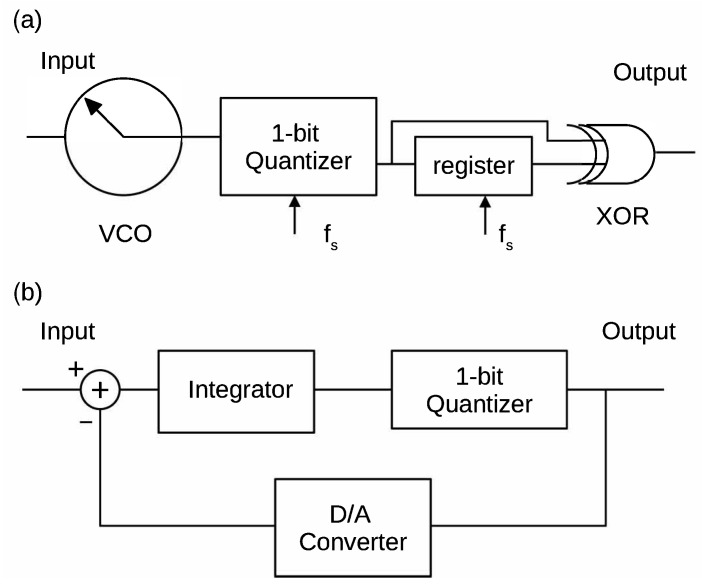
Block diagram of the delta-sigma modulators. (**a**) FDSM, (**b**) Conventional DSM.

The FDSM is a novel delta-sigma modulation technique using a VCO [4,5,7]. Figure 2 shows the configuration of the FDSM together with the conventional one. This implementation is based on the fact that the phase of the frequency modulation (FM) signal from the VCO, θ(t), is the integral of the input signal x(t), as

(1) $$\theta \left(t\right)=\int_{\tau =0}^{t}2\pi \left(f_{c}+kx\left(\tau \right)\right)d\tau .$$

**Figure 3:**
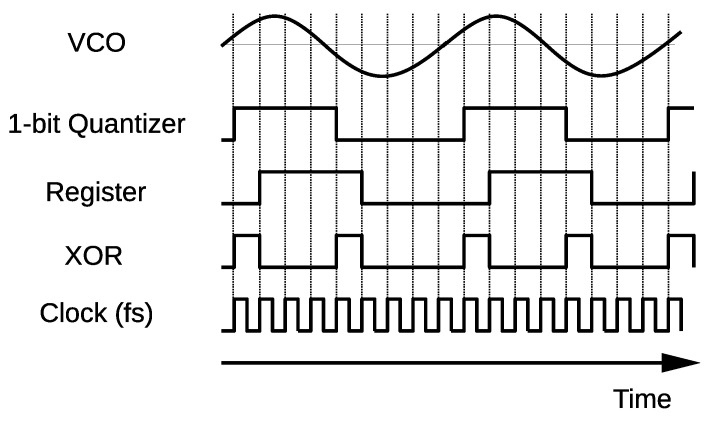
Time chart of the FDSM.

Here, $f_{c}$ represents the output frequency of the VCO when the input is zero (carrier frequency of the FM signal) and *k* the frequency sensitivity. Therefore the integrator in the conventional configuration can be removed. Moreover, the negative feedback is inherently embedded in the VCO, because the phase returns to zero when it reaches 2π (virtual feedback). Owing to these features, the feedback loop can be also removed. Using the 1-bit quantizer and the XOR, the ideal pulse density signal can be obtained. The operation of the FDSM is depicted in the time chart that is shown in Figure 3. First, the 1-bit quantizer samples the VCO output at the rising edge of the clock. It is then XOR’ed with a signal delayed by one clock cycle using the register. This means that the circuit outputs pulses when the sampled signal changes. Consequently, this circuit converts the input signal to one-bit PDM signal. It should be noted that this circuit outputs two pulses per one cycle of the VCO output. This makes the VCO frequency virtually doubled.

This technique has unique advantages. First, high frequency operation is possible, since there is no physical feedback loop. Second, the accuracy of the virtual feedback is extremely high because the feedback depends on the mathematical nature of the phase. This is in contrast with the conventional circuit, where the accuracy of the feedback is limited by the DAC in the feedback loop. Consequently, this technique is promising for wide band, high-resolution ADCs.

## 3. FDSM Microphone Sensor

The FDSM can be applied to high performance sensors when the VCO is replaced by a variable frequency oscillator whose oscillation frequency depends on a certain physical parameter. Here, we concentrate on the microphone sensor that is based on a microwave cavity resonator, which was proposed in [16].

**Figure 4:**
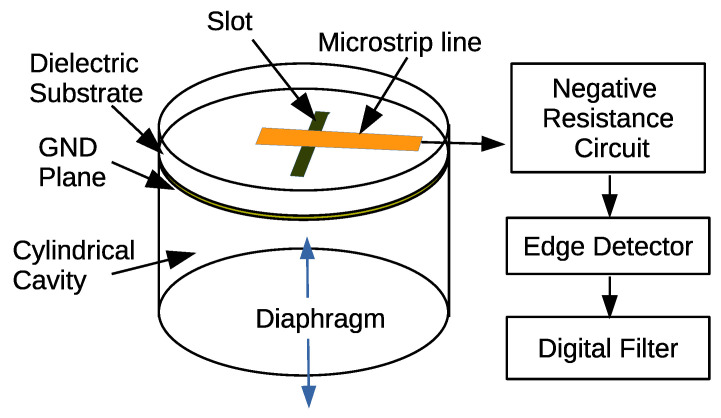
Microphone based on FDSM using a microwave cavity resonator.

Figure 4 illustrates the basic structure of the microphone. The oscillator circuit consists of a cylindrical cavity resonator and a gain block. The one end of the cavity resonator is replaced by a thin metal diaphragm, which is moved by the sound pressure. This makes the resonant frequency to change according to the sound wave. The other end is covered by the FR-4 print circuit board with a copper GND plane, where the slot is opened to couple the microstrip line to the cavity resonator. The gain block consists of a negative resistance device that is based on a gate grounded FET having a stub. A commercial heterojunction FET (Renesas Electronics, NE3514S02) was used for this circuit. This implementation is promising, because the Q-factor of the resonator is much larger than the LC oscillator using a condenser microphone and an inductor [15].

**Figure 5:**
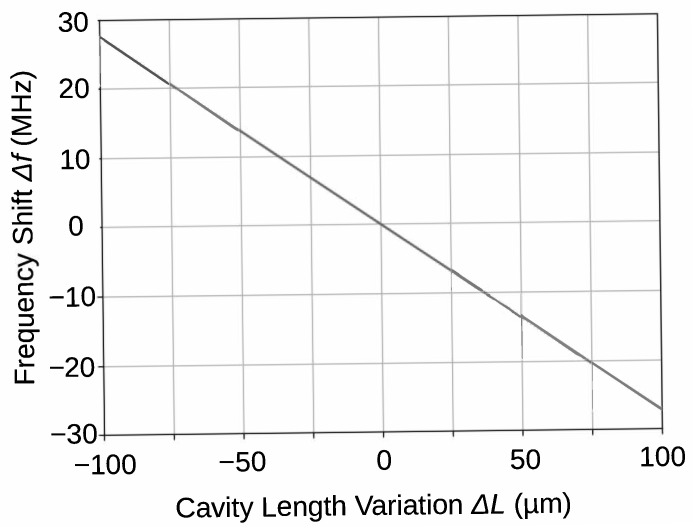
Frequency shift as a function of the cavity length variation calculated from the Equation (2).

In this report, the resonator and gain block were designed to oscillate at around 10 GHz. The size of the cavity resonator was 25 mm diameter and 20 mm length. The resonant mode was TE111, whose resonant frequency is expressed as [21]

(2) $$f_{r}=\frac{c}{2a}\sqrt{{\left(\frac{\rho_{11}^{\prime }}{\pi }\right)}^{2}+{\left(\frac{a}{L}\right)}^{2}},$$

where, $\rho_{11}^{\prime }$ is a first zero of the derivative of the Bessel function, $J_{1}$, and *c*, *a*, *L* are the speed of light, radius, and length of the cavity, respectively. The resonant frequency change, Δf, is proportional to the cavity length change, ΔL, when the change is sufficiently small. Figure 5 shows the Frequency shift as a function of the cavity length variation calculated from the Equation (2) with the above-mentioned parameters. This figure shows good linearity. The maximum non-linearity error, defined as $\max(\left|\frac{f_{\mathrm{osc}}\left(\Delta L\right)-f_{\mathrm{lin}}\left(\Delta L\right)}{f_{\mathrm{lin}}\left(\Delta L\right)-f_{\mathrm{lin}}\left(0\right)}\right|)$, is 0.6%. Here, $f_{\mathrm{osc}}\left(\Delta L\right)$ and $f_{\mathrm{lin}}\left(\Delta L\right)$ are the resonant frequency and its linear approximation at ΔL, respectively. The 100-m ΔL is sufficiently large to obtain a large dynamic range, as discussed in Section 6.4.

Regarding the membrane motion, it is the same as that in conventional condenser microphones, so that it is proportional to the sound pressure. Although the membrane is not flat under the sound pressure, it is a good approximation that the length of the cavity, *L*, is defined as a distance from the average position of the membrane to the other edge. Consequently, the sound pressure can be converted to the frequency change of the oscillator.

**Figure 6:**
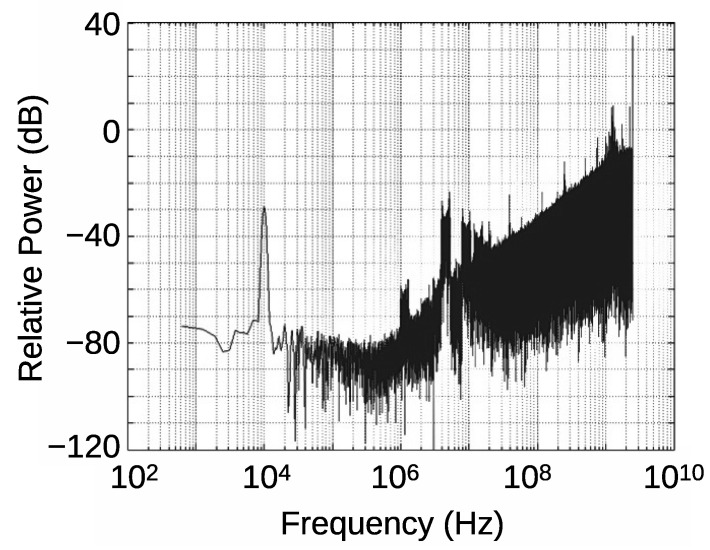
An example of the output spectrum of the delta-sigma modulation microphone [16].

Figure 6 shows a Fast Fourier Transformation (FFT) result of the output PDM signal under 10 kHz sound signal, which was reported in [16]. There are some distinct features. First, it shows clear noise shaping behavior, where the noise power decreases by 20 dB/dec when the frequency decreases. The sound signal peak is also clearly seen at 10 kHz. In addition, this figure shows the noise floor, where the noise deviates from the 20 dB/dec dependence, and it does not decreases in the frequency range smaller than 300 kHz. This degrades the SNR from that of the ideal FDSM. This noise floor must be reduced to fully exploit the performance of the FDSM sensors.

## 4. Origin of the Noise Floor

We have recently discussed the origin of the noise floor based on a simple model [20], and have shown that the phase noise of the oscillator generates this noise floor. Here, we will briefly explain the effects of the phase noise on the output spectrum of the FDSM according to the reference [20].

The phase noise is a random fluctuation of the phase of the oscillator output, and it can also be regarded as frequency fluctuation because the frequency is a derivative of the phase [22,23,24]. This fluctuation cannot be distinguished from the signal because the input signal is encoded in the FM signal in the FDSM. Consequently, such random fluctuation makes the noise floor in the output signal spectrum. The phase noise, L(f), is defined as a power per frequency that is measured at an offset frequency, *f*, from the carrier. It is normalized by the carrier power, and is usually shown in [dBc/Hz].

**Figure 7:**
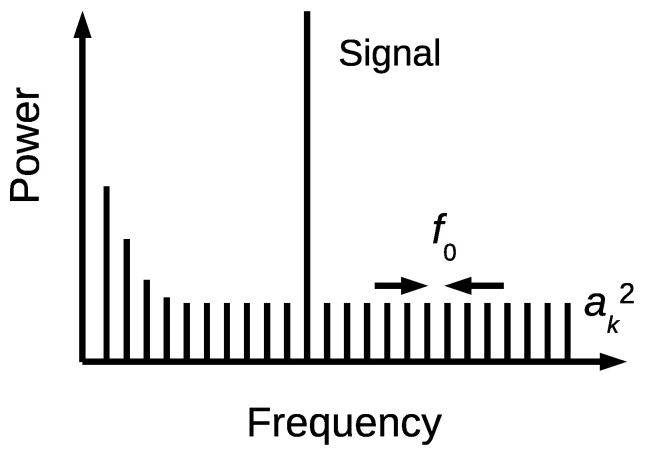
A model of the phase noise source based on uniformly distributed oscillators.

We used a simple model that is shown in Figure 7 to clarify the relation between the phase noise and the noise floor in the FDSM output. This figure shows noise sources for the variable frequency oscillator. The oscillator’s frequency is modulated by many independent oscillators with an amplitude of $a_{k}$, which are uniformly distributed in the frequency range with an interval of a frequency $f_{0}$ [25]. We used discrete representation for simplicity. Here, it is noted that the $f_{0}$ has no specific physical meaning, and this model is equivalent to the continuous spectrum, when using a sufficiently small $f_{0}$. Here, a sinusoidal input signal is assumed and also shown in the figure. The flat white noise component generates $f^{-2}$ noise, and the low frequency excess noise generates $f^{-\alpha }$ noise (α>2) in L(f).

We assume that the sampling frequency is so high that the output of the FDSM can be approximated by a continuous pulse density function PD(t), as

(3) $$\mathrm{PD}\left(t\right)=\frac{f_{\mathrm{osc}}\left(t\right)}{f_{s}/2}=\frac{2f_{\mathrm{osc}}\left(t\right)}{f_{s}}.$$

Here, $f_{\mathrm{osc}}\left(t\right)$, $f_{s}$ are the instantaneous frequency of the oscillator and the sampling frequency, respectively. Under this assumption, it is shown that the noise of power density $a_{k}^{2}/\left(2f_{0}\right)$ per Hz is added to the FDSM signal at the frequency, $kf_{0}$. In order to compare this with the quantization noise, we normalized this noise by the full-scale range, $f_{s}/4$. By using the relation between L(f) and $a_{k}$, we obtained an expression below. The details of the derivation are shown in reference [20].

(4) $$p_{\mathrm{ph}}\left(f\right)=\frac{64f^{2}L\left(f\right)}{f_{s}^{2}}.$$

This equation relates the phase noise, L(f), to the noise floor of the FDSM output, $p_{\mathrm{ph}}\left(f\right)$. It should be noted that the frequency *f* in this equation is a frequency of the output spectrum. It means that the offset frequency of the phase noise L(f) directly corresponds to that of the output spectrum.

On the other hand, the normalized quantization noise power density, $p_{Q}\left(f\right)$ can be expressed as [2,3,26],

(5) $$p_{Q}\left(f\right)=\frac{16}{3f_{s}}\sin^{2}\left(\frac{\pi f}{f_{s}}\right)≃\frac{16\pi^{2}f^{2}}{3f_{s}^{3}}.$$

The same normalization scheme ensures the direct comparison of the phase noise and the quantization noise.

In addition, the phase noise of the sampling clock, $L_{s}\left(f\right)$, was also analyzed by the similar model. The pulse density function PD(t), including frequency fluctuation of both the carrier frequency, $f_{c}$, and the sampling clock, $f_{s}$, can be expressed as

(6) $$\mathrm{PD}\left(t\right)=2\frac{f_{c}+\Delta f_{c}}{f_{s}+\Delta f_{s}}≃\frac{2}{f_{s}}\left(f_{c}+\Delta f_{c}-\frac{f_{c}}{f_{s}}\Delta f_{s}\right).$$

Here, $\Delta f_{c}$ and $\Delta f_{s}$ are the fluctuations of the carrier and sampling clock frequencies, respectively. This indicates that the contribution of $\Delta f_{s}$ is smaller than that of the $\Delta f_{c}$ by a factor of $\left(f_{c}/f_{s}\right)$. The noise power density due to the phase noise of the sampling clock was thus obtained as

(7) $$p_{\mathrm{phs}}\left(f\right)=\frac{64f^{2}L_{s}\left(f\right)}{f_{s}^{2}}{\left(\frac{f_{c}}{f_{s}}\right)}^{2}.$$

This indicates that the lower carrier frequency is advantageous for suppressing the phase noise effects of the sampling clock. However, in the general case, higher oscillation frequency leads to larger frequency modulation width, which enhances the sensitivity. Therefore, it is advantageous to down-convert high frequency FM signal before sampling.

## 5. Cavity Resonator Oscillators with Lower Phase Noise

It is important to reduce the phase noise of the variable frequency oscillator for realizing high performance FDSM microphone sensors, as described in the previous section. Here, we will discuss the design and fabrication of low phase noise oscillators for these sensors.

### 5.1. Cavity Resonators

One of the most important parameters, which dominates the phase noise, is a Q-factor of the resonator. In the previous paper [16], the Q-factor of the resonator was approximately 700. This is comparatively low value for the cylindrical cavity resonator. This low value is attributed to the material and the surface roughness of the resonator. Subsequently, here we used low resistivity oxygen-free copper for the resonator instead of the brass used in [16]. The inside of the resonator was polished and cleaned by chemical cleaner. We fabricated the cavity resonators having the same diameter of 25 mm, and the length was varied from 20 to 25 mm. Subsequently, the resonator Q-factors were measured using a vector network analyzer. Here, the thin diaphragm was replaced by a thick copper plate to measure the intrinsic characteristics of the resonator. In this paper, we used the two-port method, because the transmission type oscillator topology (parallel feedback) was used, as described in the next subsection.

**Figure 8:**
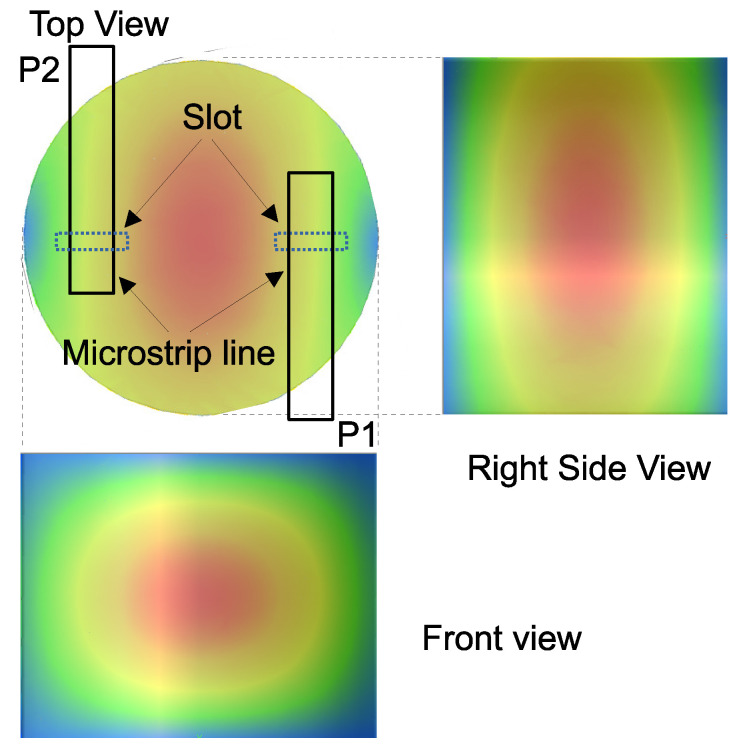
Electric field strength in the cavity resonator for TE111 mode.

Figure 8 shows the electric field distribution of the TE111 mode that was calculated by electromagnetic FEM simulation together with the microstrip lines on the top view. The top of cavity was covered by the 1.6 mm FR-4 print circuit board with a copper GND plane, where the slots were opened under two microstrip lines. The size of the slot was 1×4 mm${}^{2}$. The width of the microstrip line was 2.9 mm, which corresponds to 50 Ω impedance. The distance between two microstrip lines was 16 mm (center-to-center), and the distance from the slot to the open end of the microstrip line is 4.2 mm, which works as a λ/4 open stub. These stubs ensure maximum current at the slot, and the magnetic field that is generated by these currents couple with the magnetic field in the cavity, which coils around the electric field that is shown in the figure.

**Figure 9:**
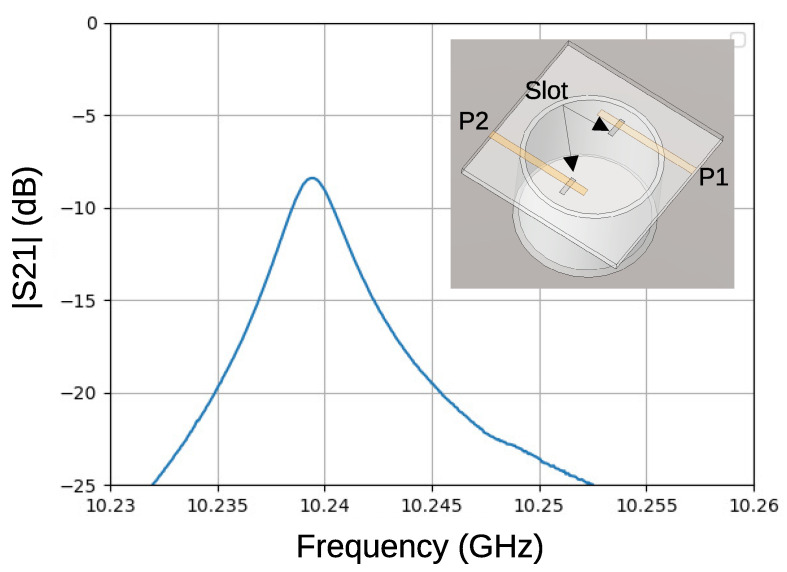
S21 of the oxygen-free copper based resonator.

Figure 9 shows an example of the magnitude of the transfer characteristics, |S21|, of the resonator (the cavity length is 20 mm). There is a sharp peak at approximately 10.24 GHz, which agrees well with the frequency that was calculated by the FEM simulation. A high loaded Q-factor, $Q_{L}$ of 3660 was calculated from the FWHM of the peak. Using the equation,

(8) $$Q_{U}=\frac{Q_{L}}{1-\left|S21\right|}$$

the unloaded Q-factor, $Q_{U}$, is also estimated to be 5900. Similar results were obtained for the resonators having different lengths. These values are much larger than those of the previous one, and advantageous for low phase noise.

### 5.2. Oscillator Circuit Design

An oscillator has two-types of topologies, which determine how the amplifier and resonator are coupled in a feedback arrangement [27]. Our previous report [16] used the “reflection” type topology (negative resistance oscillator, or series feedback oscillator). This topology is simple and widely used for microwave/millimeter wave oscillators; however, it has the drawback that a number of important parameters like resonator loading, output power and amplifier compression are tightly coupled and hard to control separately. On the other hand, transmission type oscillator (parallel feedback oscillator) gives much better control of the critical parameters. Therefore, we used transmission type oscillator topology for improving the phase noise property.

**Figure 10:**
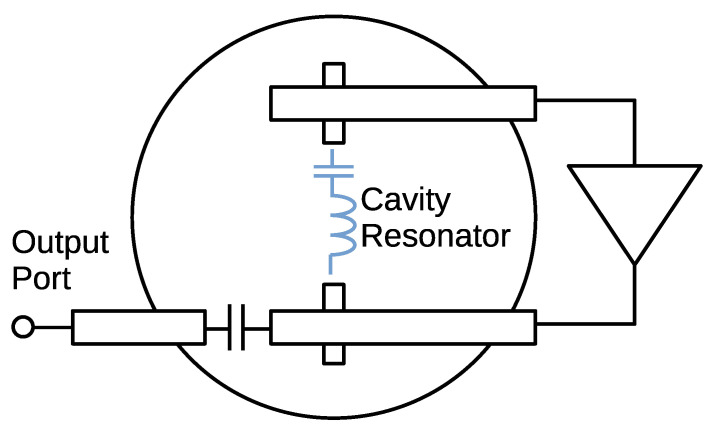
Oscillator circuit used for the sensor.

**Figure 11:**
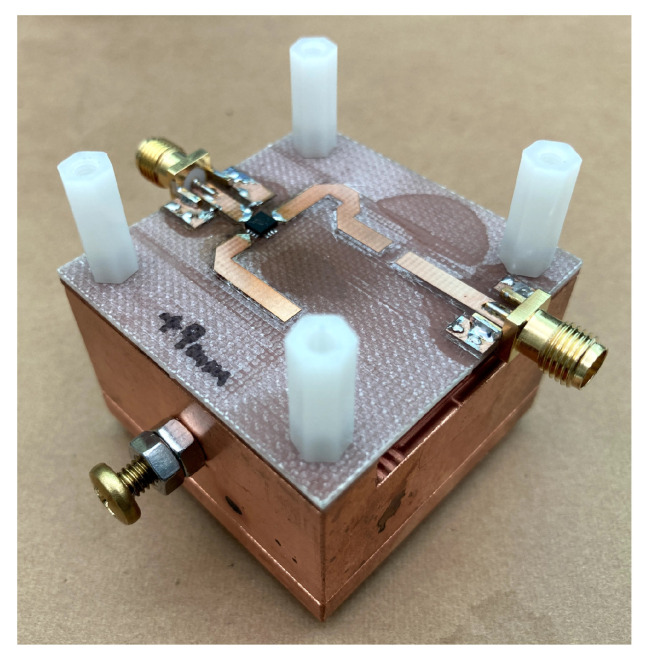
Fabricated oscillator/sensor.

Figure 10 and Figure 11 show the circuit diagram and the photograph of the oscillator that we fabricated. We used a GaAs pHEMT MMIC medium power amplifier, HMC441LP3E (Analog Devices), as a gain block of the oscillator. The width of the microstrip line was 2.9 mm corresponding to 50 Ω impedance. The output port was coupled to one of the open end of the microstrip line with a small capacitance. For Barkhausen’s condition to suffice, the lengths of the microstrip lines were designed to ensure the phase shift around the loop is zero. The most important parameter to determine the phase noise is the degree of coupling to the resonator. For an ideal case, when the thermal noise is the major noise source, it was shown that the coupling should be $Q_{L}=Q_{U}/2$, which corresponds to the |S21| of −6 dB at the resonant frequency (|S21|=1/2) [28,29]. However, in our case, weaker coupling is advantageous for the phase noise property. This is because the flicker noise cannot be ignored, and it is converted to the oscillator phase noise by the non-linearity of the amplifier. To suppress the non-linearity, the loop loss should be increased to weaken the compression of the amplifier output. We chose |S21| of around −10 to −12 dB, because the gain of the amplifier is 14 dB. This also increases the loaded Q.

**Figure 12:**
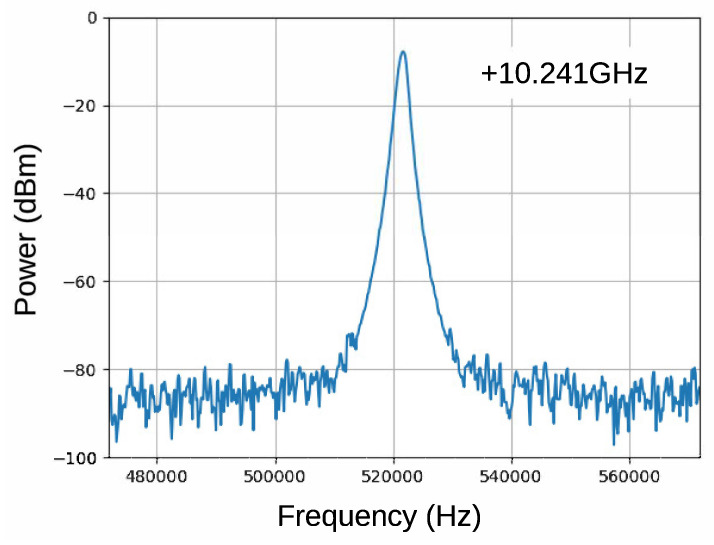
Output spectrum. Frequency span is 100 kHz with 10.241 GHz offset.

**Figure 13:**
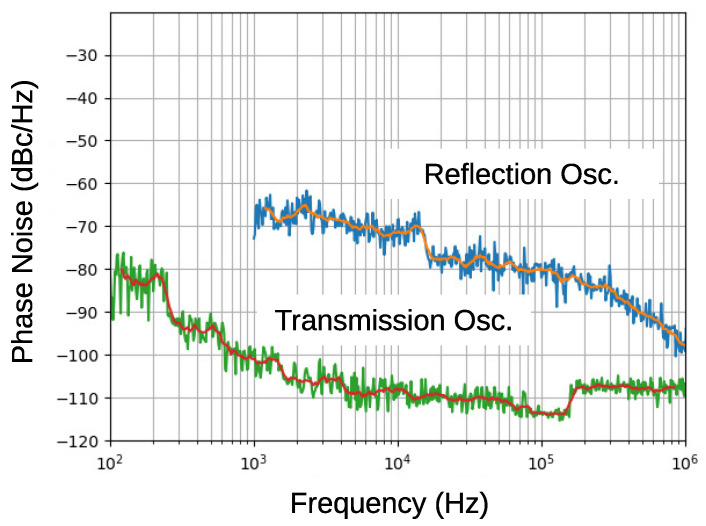
The phase noise of the fabricated transmission oscillator with that of the reflection oscillator. Moving averages are also plotted in the figure.

The fabricated oscillator oscillated at 10.241 GHz, which agrees well with the resonant frequency. Figure 12 presents the output spectrum. A sharp and stable oscillation was observed. Phase noise, shown in Figure 13, was measured using Keysight 8565EC spectrum analyzer. The phase noise of the previous paper, the result of the reflection type oscillator, is also shown in the figure for comparison. It should be noted that the minimum value of the transmission oscillator is limited by the noise floor of the spectrum analyzer (−117 dBc/Hz), so that it might be still smaller at frequencies larger than 10 kHz. A significant phase noise reduction of more than 30 dB was achieved by improved circuit design and cavity resonator. This leads to noise floor reduction in FDSM sensors.

## 6. Experimental Results of the FDSM Microphone Sensors

### 6.1. FDSM Analyzer Circuit on an FPGA

**Figure 14:**
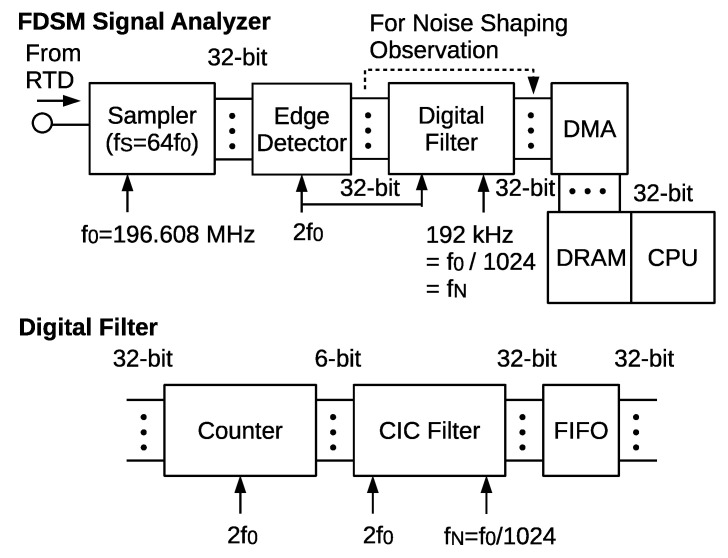
Block diagram of the signal-analyzer circuit on an FPGA. The lower illustration details the digital filter, which eliminates the high-frequency noise components and converts the PDM signal to a 32-bit parallel signal at the Nyquist rate.

We have fabricated an FDSM signal analyzer circuit on a field programmable gate array (FPGA) board, Xilinx ZCU-102 (XC7K325T-2FFG900C) [19]. Figure 14 shows its block diagram. It comprises a high-frequency sampler, an edge detector, and a digital-filter. A high-frequency transceiver module was used for the sampling circuit, which could be operated at the sampling rate of as high as 16.3 Gb/s. Here, the sampling rate was chosen to be 12.582912 Gb/s, which corresponds to the oversampling ratio of 65,536 for a signal bandwidth of 96 kHz.

The sampled 1-bit digital signal was converted to a 32-bit parallel data stream and then fed to the edge detector. This edge detector consists of XORs and registers, and works similarly to Figure 3, but in a parallel manner. Subsequently, the 32-bit PDM data stream was compressed to a 6-bit data stream by counting “1”. This works as a moving average LPF. Next, the 6-bit data stream was fed to the cascaded-integrator-comb (CIC) filter, which functioned as a sinc2 filter. The filter module is shown in the lower illustration in Figure 14. The output data were transferred to the random access memory, and displayed on a PC. When we tested the noise shaping property, the filter was bypassed.

### 6.2. Experimental Setup

**Figure 15:**
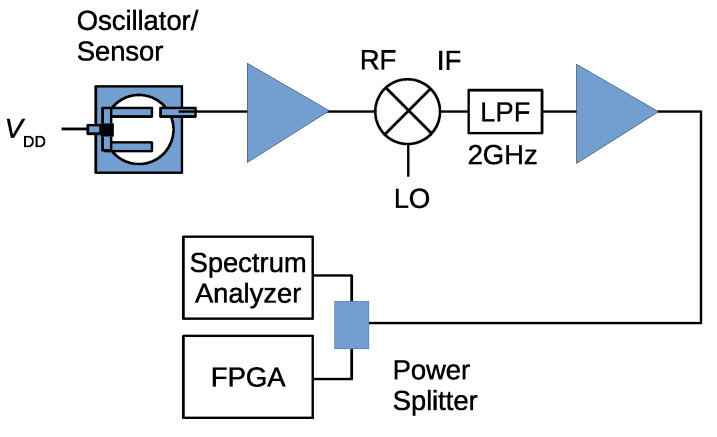
Experimental setup for noise floor measurement and sound detection.

The effects of the sampling clock phase noise is a function of the carrier frequency of the FM signal, and it decreases by −20 dB/dec, as mentioned in Section 4. Therefore, we decided to down-convert the FM signal from the oscillator/sensor. Figure 15 shows the experimental setup used for the noise floor measurement and also for the sound detection. The output of the oscillator/sensor was amplified and fed to the mixer (Mini-Circuit ZX05-153MH-S+). The down-converted IF signal was filtered by 2 GHz LPF and amplified. Finally, it was fed to the FPGA or spectrum analyzer.

### 6.3. Noise Floor and Total in-Band Noise Power

**Figure 16:**
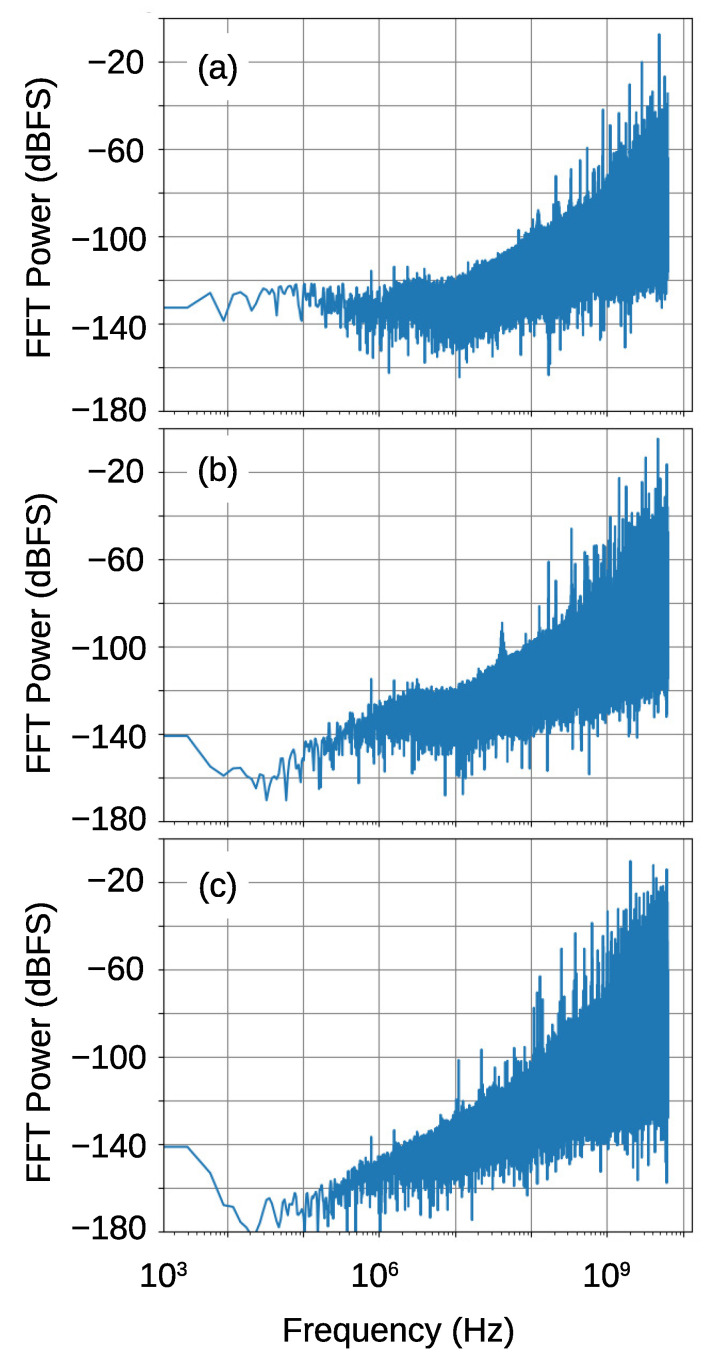
FFT spectra of the output PDM signals from the FPGA. The top figure (**a**) shows the result of the reflection oscillator in the previous work. The second figure (**b**) shows the result of the transmission oscillator, and the bottom figure (**c**) shows that of the transmission oscillator when the signal was down-converted by the mixer with 9.2 GHz LO. The results are normalized by the full-scale input, $f_{s}/4$.

Figure 16 shows the FFT results of the PDM signals. The top figure shows the result of the reflection oscillator in the previous work. It shows large noise floor in the frequency range less than 10 MHz at around −130 dBFS. The second figure shows the result of the transmission oscillator that was fabricated in the present work. Although the noise floor is considerably reduced from the top figure, there is a step like structure at around 10 MHz. This should be due to the phase noise of the sampling clock. This structure was disappeared in the bottom figure where the output signal from the transmission oscillator was down-converted by the mixer with 9.2 GHz LO.

**Figure 17:**
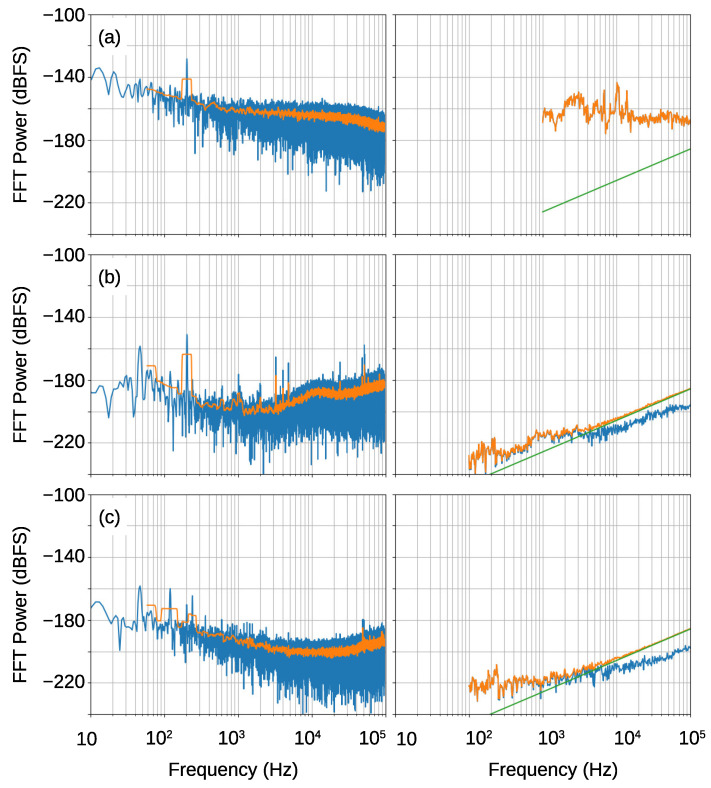
Measured and calculated in-band noise spectra. **Left** figures: measured results from the FDSM analyzer, **Right** figures: calculated noise spectra. In the left figures, the moving averages are plotted by orange lines. In the right figures, the blue lines show the noise due to the phase noise, the green lines show the quantization noise, and the orange lines show the sum of two noises. (**a**) Reflection oscillator with down-conversion, (**b**) transmission oscillator without down-conversion, (**c**) transmission oscillator with down-conversion. In the right figure in (**a**), the total noise is almost equal to the noise due to the phase noise. Consequently, the blue line is completely overlapped by the orange line.

Next, we investigate the details of the in-band noise properties. Figure 17 shows the measured FDSM noise floors (left figures) with those that are calculated using the Equations (5) and (7) (right figures). In the right figures, the blue lines show the noise due to the phase noise, the green lines show the quantization noise, and the orange lines show the sum of two noises.

The top figures show the result of the reflection type oscillator that was reported in [16]. Here, the output of the oscillator was down-converted to 1 GHz with an LO signal from a signal generator, Keysight 83650L. The noise floor is very high due to the large phase noise that is shown in Figure 13. The calculated noise floor using Equation (7) agrees well with measured result. It should be noted that the noise floor that is caused by the phase noise is much larger than the quantization noise. This indicates that the signal-to-noise ratio (SNR) and dynamic range (DR) were dominated by the phase noise, and one cannot increase them even though the sampling frequency is increased.

The middle and bottom figures show the results of the transmission oscillator that we fabricated here. The output of the oscillator is directly fed to the FPGA in the middle figures, and the down-converted signal using the another transmission oscillator (9.2 GHz output) is used for the bottom figure. The direct sampling result shows larger noise floor due to the phase noise of the sampling clock. On the other hand, the bottom figures show smaller noise. The measured result agrees well with the calculated noise at frequencies that are higher than about 10 kHz. We suspect that the deviation at lower frequencies should be caused by the environmental vibration noise, especially through cables, although further studies are needed to clarify the origin. A most important point is that the total noise is dominated by the quantization noise. This indicates that a further reduction of the noise floor can be obtained when the sampling clock frequency is increased.

Table 1 summarizes the total noise power in the signal bandwidth. A large reduction of the noise can be achieved by an improved Q-factor and oscillator design. The most interesting point of this table is that the lower total noise is obtained than the ideal quantization noise for the transmission oscillator with down-conversion. This is because the theoretical model averages the pattern noise [2,3,30]. If the carrier frequency is chosen so that the pattern noise is small, the quantization noise can be less than the theoretical value. This is advantageous for an application with a high oversampling ratio and a narrow frequency modulation [30].

### 6.4. Sound Spectra

In this subsection, we describe the response of the sensors to sound pressure. The thick copper plate at the end of the cavity was replaced by a 12 μm-thick Al foil as a diaphragm. A calibrator for a sound meter was used for the sound source.

**Figure 18:**
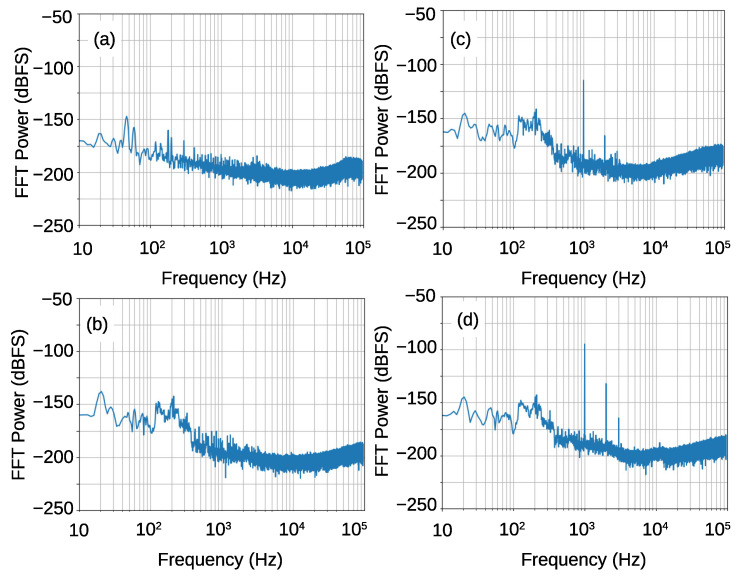
Sound spectra from the FDSM microphone sensors. (**a**) Covered by 5 mm Copper plate in place of the calibrator, (**b**) No sound signal, (**c**) under 1 kHz, 94 dBSPL sound signal, (**d**) under 1 kHz 114 dBSPL sound signal. The FFT results were averaged for three data series.

Figure 18 shows the spectra of the output 32-bit digital signal at 192 kHz. Figure 18a shows the result when the diaphragm was covered by thick copper plate, so that no sound pressure is applied to the diaphragm. No noticeable change was observed from the result without the Al foil diaphragm shown in Figure 17c. This indicates that the Q-factor does not change, even when the copper plate is changed to the Al-foil diaphragm. The in-band total noise was −144 dBFS.

The Figure 18b–d show the results when the calibrator was fixed to the diaphragm frame, and hermetically sealed with vacuum grease. Figure 18b shows the result when the calibrator was switched off. Because of the environmental vibration and sound noise, larger noise power than that in Figure 18a was observed at frequencies less than 500 Hz. Figure 18c,d show the spectra under 1 kHz-sound pressure of 94 dBSPL and 114 dBSPL, respectively. We used Blackman–Harris window function for the FFT, so that the peak was somewhat broadening. The integrated power of the sound peak at 1 kHz was −106.5 and −87.0 dBFS for 94 and 114 dBSPL sound pressures, respectively. The peak power difference agrees well with the input power difference, which indicates good linearity. The SNR was calculated to be 37.5 and 57 dB for 94 and 114 dBSPL input, respectively, because the intrinsic in-band noise power was −144 dBFS.

It is interesting to mention that the total noise power was −154 dBFS when the band width is limited to 20 kHz. The difference to that for 96 kHz bandwidth was 10 dB, which was larger than 10log(96 k/20 k)∼6.8 dB. This is because the noise shaping behavior also dominates the total noise at frequencies that are less than 96 kHz. The SNR for 20-kHz bandwidth was 47.5 and 67 dB for 94 and 114 dBSPL, respectively.

A most important advantage of the FDSM microphone is a wide dynamic range (DR). The DR for this device is estimated to 87 dB for maximum sound pressure of 140 dBSPL. Non-linearity is expected to be sufficiently small even for this large power, because it is still much smaller than full-scale power (∼−60 dBFS). This DR is still limited by a small frequency modulation in this device. The peak-to-peak frequency modulation width for 114 dBSPL was approximately 200 kHz. This corresponds to the peak-to-peak diaphragm motion of about 0.8 μm. This also degrades the minimum measurable sound power, which is 47 dBSPL for 20 kHz bandwidth. This is much larger than that of today’s high performance microphones, ≲15 dBSPL. This is due to the thick, 12 μm, Al diaphragm, which restricts the amplitude of the diaphragm motion. The thickness of the diaphragm can be reduced to the microwave skin depth of the oscillator’s frequency. The skin depth at 10 GHz is around 0.8 μm for Al. More than 20 dB reduction of the minimum measurable sound power is expected. Moreover, the noise floor is now dominated by the quantization noise, as shown in Figure 17c. Therefore, it can be reduced when increasing the sampling frequency, or equivalently, using parallel sampling. Consequently, 40 dB improvement is expected. In addition, because the performance of the FDSM sensors depends on the oscillation frequency, further improvement is expected when reducing the size of the device.

## 7. Conclusions

Drastic in-band noise reduction in FDSM microphone sensors was demonstrated by improving the phase noise of the oscillator. First, to increase the Q-factor of the cavity resonator, we improved the material and surface treatment condition, and obtained a large unloaded Q-factor of around 6000. Next, we employed the transmission type oscillator circuit instead of the reflection type oscillator used in the previous report, because this topology gives much better control of the critical parameters. With these improvements, the phase noise was improved by approximately 40 dB. Finally, we tested the microphone sensors based on these improved oscillators and demonstrated the noise floor reduction of around 40 dB. In addition, we obtained an SNR of 57 dB for 114 dBSPL sound input and 96 kHz bandwidth, which corresponds to 87 dB DR. A much larger DR of around 120 dB is expected by increasing the sampling rate and decreasing the Al diaphragm thickness.

## Figures and Tables

**Table 1:** In-band total noise power.

Sample	Measured	Calculated (dBFS)		
	(dBFS)	Total	Quantization Noise	Phase Noise
Ref. Osc. down-conv.	−114	−112	−141	−112
Trans. Osc. direct	−133	−141	−141	−150
Trans. Osc. down-conv.	−143	−141	−141	−152

## Abbreviations

The following abbreviations are used in this manuscript:

ADC	Analog-Digital Converter
DSM	Delta-Sigma Modulator
PDM	Pulse Density Modulation
DAC	Digital-Analog Converter
FDSM	Frequency Delta-Sigma Modulator
FM	Frequency Modulation
VCO	Voltage Controlled Oscillator
FET	Field Effect Transistor
FFT	Fast Fourier Transform
FEM	Finite Element Method
FWHM	Full Width of Half Maximum
pHEMT	pseudomorphic High Electron Mobility Transistor
FPGA	Field Programmable Gate Way
CIC	Cascaded Integrator-Comb
LO	Local Oscillator
SNR	Signal-to-Noise Ratio
DR	Dynamic Range
dBSPL	decibel sound pressure lebel
dBFS	decibel full-scale
